# Study of electrical properties of cobalt oxide-doped sodium meta vanadate at different frequencies

**DOI:** 10.1016/j.heliyon.2022.e10471

**Published:** 2022-08-30

**Authors:** Rasha Hosam Saleh, Mohammad Deeb

**Affiliations:** Chemistry Department, Faculty of Sciences, Tishreen University, Lattakia, Syria

**Keywords:** Sodium meta vanadate NaVO_3_, Cobalt oxide Co_3_O_4_, Specific electrical resistivity **ρ**, Electrical capacitance C, Relative dielectric constant Ԑ_r_, Loss tangent tanδ

## Abstract

The samples were prepared within the binary system NaVO_3_–Co_3_O_4_ at different molar percentages depending on the ceramic method according to _(x)_NaVO_3_−_(100-x)_Co_3_O_4_, for the molar percentages (x = 5, 10, 25, 40, 60, 75, 80) mole%. The prepared samples were studied using X-rays diffraction (XRD), and their electrical properties were studied using LCR meter, such as the specific electrical resistivity **ρ**, electrical capacitance C, relative dielectric constant Ԑ_r,_ and loss tangent tanδ within the frequency range (10Hz-MHz). The results showed that the resistivity values of all the prepared samples increased, and the highest value is 2.91 × 10^+07^ (Ω.cm), in addition to the increase in the values of electrical capacitance and the highest value is 9.05 pF, the highest value of the relative dielectric constant is 28.39, and the highest value of the loss tangent is 7.01 × 10^−2^, with the change in the molar percentage of sodium meta vanadate in the samples.

## 1 Introduction

The search for materials and chemical compounds that play the largest role in modern technical applications is the basis of scientific studies and current research trends. Cobalt oxide Co_3_O_4_ is considered an electrically important compound, especially when it is used in batteries, as it is characterized by high capacity and high density, in addition to its stable chemical properties [1]. which makes it have important advantages such as high energy density, low cost, in addition to being environmentally friendly [2]. Also, crystalline sodium meta vanadate NaVO_3_ has a great importance in electrical insulation [3]. It is used as a cathode in batteries such as a sodium ion battery in which the charge can be maintained for a long time and the charge transfer during charging and discharging can be compensated due to the unique behavior shown by sodium meta vanadate NaVO_3_ [4].

Despite the multiplicity of the used chemicals in batteries, many recent studies have focused on cobalt oxide Co_3_O_4_, as its qualities such as high electrical capacity and low cost lead to ideal performance in many applications [5]. Interest has increased when it is used in high-performance lithium/sodium-ion batteries due to their improved safety, high volumetric energy density, and superior storage capacity [6]. In addition to its ability to improve the electrochemical properties when it is used as a cathode material for lithium-ion batteries [7]. Its application was studied using X-ray diffraction (XRD), scanning electron microscope (SEM), transmission electron microscope (TEM), and Raman spectra, all of which confirmed its great importance [8].

Sodium meta vanadate NaVO_3_ has also been used in many types of batteries, which has shown to improve their characteristics and performance [9]. As it increases the capacity of batteries to store energy, extending their life, and helping to increase their reversibility and enhance their electrochemical performance [10].

### 1.1 The importance and objectives of the research

This research is considered one of the research that takes a large space in the current scientific studies, which focus on artificial intelligence and technological development, especially in the field of batteries in order to improve their specifications. The research aims to:

• obtain different phases of solid compounds within the binary system NaVO_3_–Co_3_O_4_ that have important electrical specifications that can be used in the field of batteries, supercapacitors and solar energy cells from raw materials of great importance in this field. In addition to studying the difference of their properties according to the prepared molar percentages.
• Focus on the economic aspects of obtaining these solid phases at a law cost, and within easy-to-apply terms.

## 2 Materials and research methods

### 2.1 Materials

- Sodium meta vanadate NaVO_3(S)_ with a purity of 99%− Geel, Belgium company.
- Cobalt oxide Co_3_O_4(S)_ with a purity of 99%− Bhiwadi company

### 2.2 Sample preparation

Depending on the ceramic method, samples were prepared from powdered raw materials NaVO_3,_ Co_3_O_4,_ Within the binary system _(x)_ NaVO_3_−_(100-x)_Co_3_O_4_ [11]. The weights of the used materials were calculated for the following molar percentages (x = 5, 10, 25, 40, 60, 75, 80), as shown in Table 1:

**Table 1:** The method of calculating the proportions taken from each raw material by the proportion dependency x.

x	composition	unit wt	
		NaVO_3_	Co_3_O_4_
5	5% NaVO_3_ + 95% Co_3_O_4_	0.0260	0.9740

Then the samples were prepared according to the following steps:

The material was sieved using a manual sieve to obtain the same size of particles. Depending on the molecular weights of the materials NaVO_3_ and Co_3_O_4_, the raw materials were weighed using a digital lab weighting scale (0.0001 g), according to the formula _(x)_ NaVO_3_−_(100-x)_Co_3_O_4_ by proportions (x = 5, 10, 25, 40, 60, 75, 80) mole%. The two processes of grinding and manual mixing were well carried out in an agate mortar for (7–8) hours for each sample. Then samples were prepared in the form of tablets by a manual hydraulic press with a capacity of 3 ton/cm^2^. After that the samples were heated in an incinerator furnace (Carbolite, BAMFORD, SHEFFIEJD, ENGLAND, S30 2 AU) at 150 °C for 8 h. The manual grinding and mixing processes, then pressing and heating were repeated by the same mechanism within the range (150–550)°C, with the temperature increasing 25° each time until it reaches 550 °C. Then it was sintered at 550 °C for 15 h. Then the diameter and thickness were measured by means of a digital vernier caliper as shown in Chart 1.

**Chart 1:** the diameter and thickness of the tablets.

Molar percentage of tablet (x)	Tablet Thickness d (cm)	Tablet Surface Area A (cm)^2^
5	0.345	1.8337
10	0.349	1.8337
25	0.358	1.8337
40	0.402	1.8337
60	0.54	1.8337
75	0.509	1.8337
80	0.317	1.8337

### 2.3 Study samples

The prepared samples were studied with XRD diffraction, using rays from copper metal [λ Kα_1_] = 1.54060^o^A. And LCR (PM 6306) meter device, the Intensive suppository diameter is 1 cm.

## 3 Results and discussion

### 3.1 XRD diffraction

**Figure 1:**
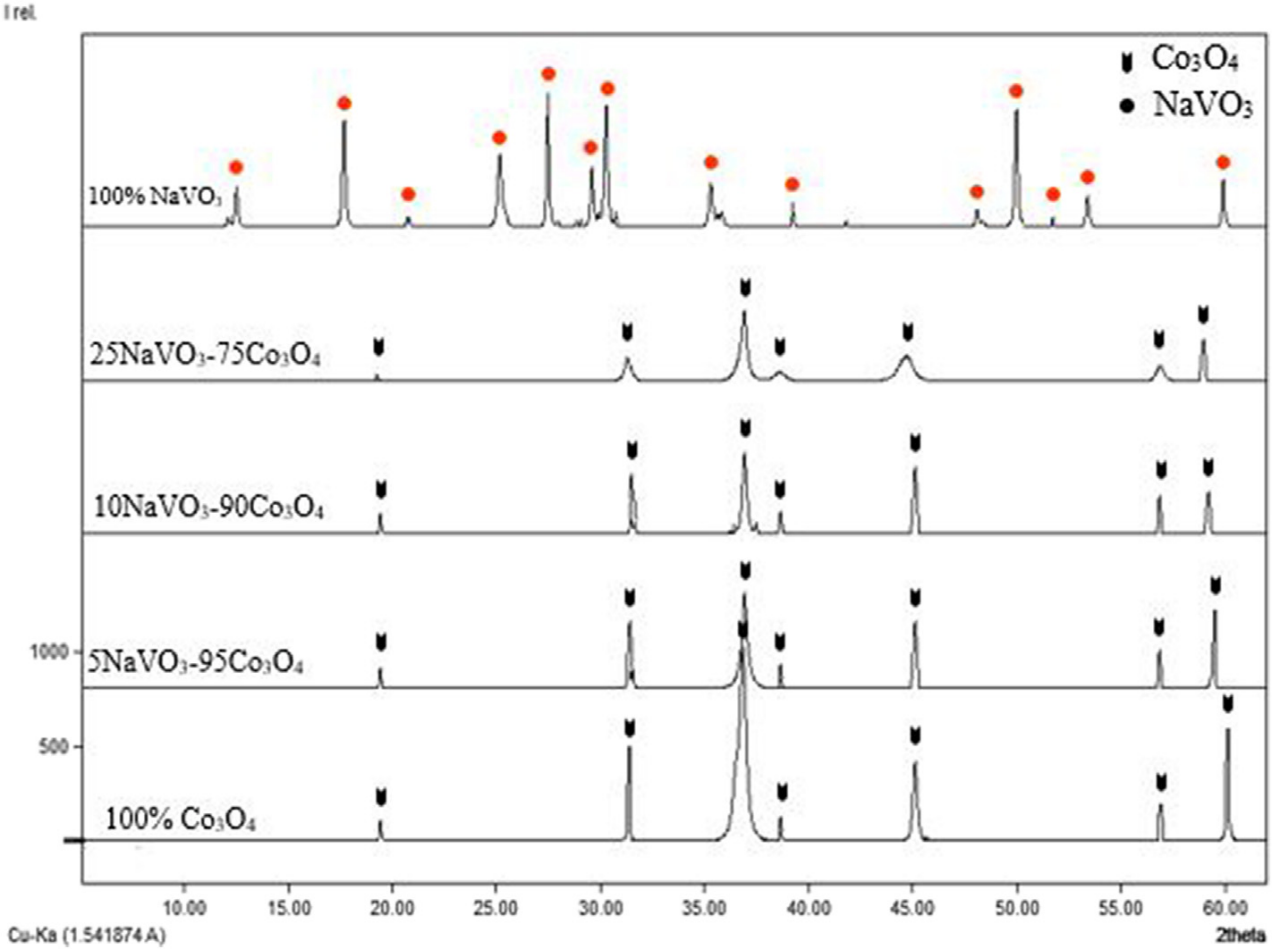
Spectra of a crystalline solid solution formed on the basis of cobalt oxide.

**Figure 2:**
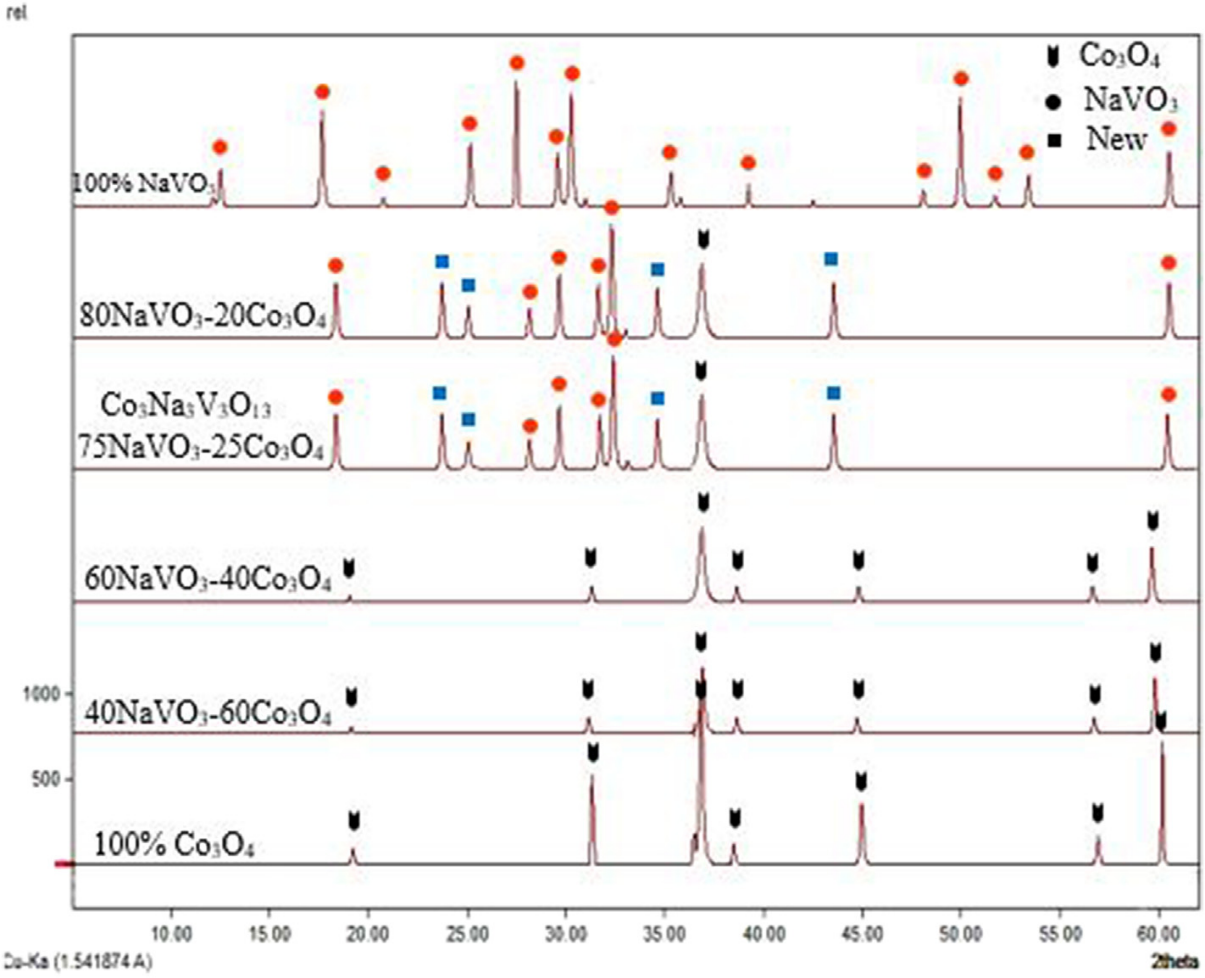
The new crystalline compound and the solid solution formed on its basis.

The prepared samples within the binary system NaVO_3_–Co_3_O_4_ were studied according to the molar percentages mentioned previously, in addition to the raw materials. Where sodium meta vanadate has the monoclinic crystal system, and cobalt oxide has a cubic crystal system. After comparing the resulting spectra of samples with the spectra of the raw materials, the results showed the following:

At the molar percentage 100% Co_3_O_4,_ all peaks belong to the pure cobalt oxide compound. The cobalt oxide spectrum and its characteristic peaks continue until the molar percentage 60NaVO_3_─40Co_3_O_4_ mole%. Which indicates the formation of a crystalline solid solution on the basis of the cobalt oxide compound up to this molar percentage, due to the dissolution of sodium meta vanadate in cobalt oxide, as shown in Figures 1 and 2:

At the molar percentage 100% NaVO_3_, all peaks belong to pure sodium meta vanadate compound. We note that at the molar percentage of 75NaVO_3_─25Co_3_O_4_ mole%, almost all the cobalt oxide peaks disappear, The emergence of some peaks of meta-sodium vanadate, in addition to the emergence of other new peaks. Which indicates the formation of a new crystalline compound with a different spectrum from the spectrum of the raw used materials, its chemical formula Co_3_Na_3_V_3_O_13_. It is produced by the reaction of sodium meta vanadate with cobalt oxide, as shown in Figure 2.

The spectrum of the new crystalline compound Co_3_Na_3_V_3_O_13_ continues from _80_NaVO_3_─_20_Co_3_O_4_ mole%. Which indicates the formation of a crystalline solid solution on the basis of the crystalline compound Co_3_Na_3_V_3_O_13_. It is caused by the dissolution of sodium meta vanadate in the new crystalline compound Co_3_Na_3_V_3_O_13_, as shown in Figure 2:

### 3.2 Effect of the molar ratio of NaVO_3_ on the specific electrical resistivity ρ

The specific electrical resistance (Ω.cm) was studied for prepared samples according to the binary system _(x)_NaVO_3_−_(100-x)_Co_3_O_4_ Within the frequency range (10Hz-1MHz). The diameter of the Intensive suppository is 1 cm, using the relationship $\text{ρ}=\text{R}\;\frac{A}{d}$, where R: electrical resistance (Ω), A: tablet surface area (cm)^2^, d: tablet thickness (cm).

**Figure 3:**
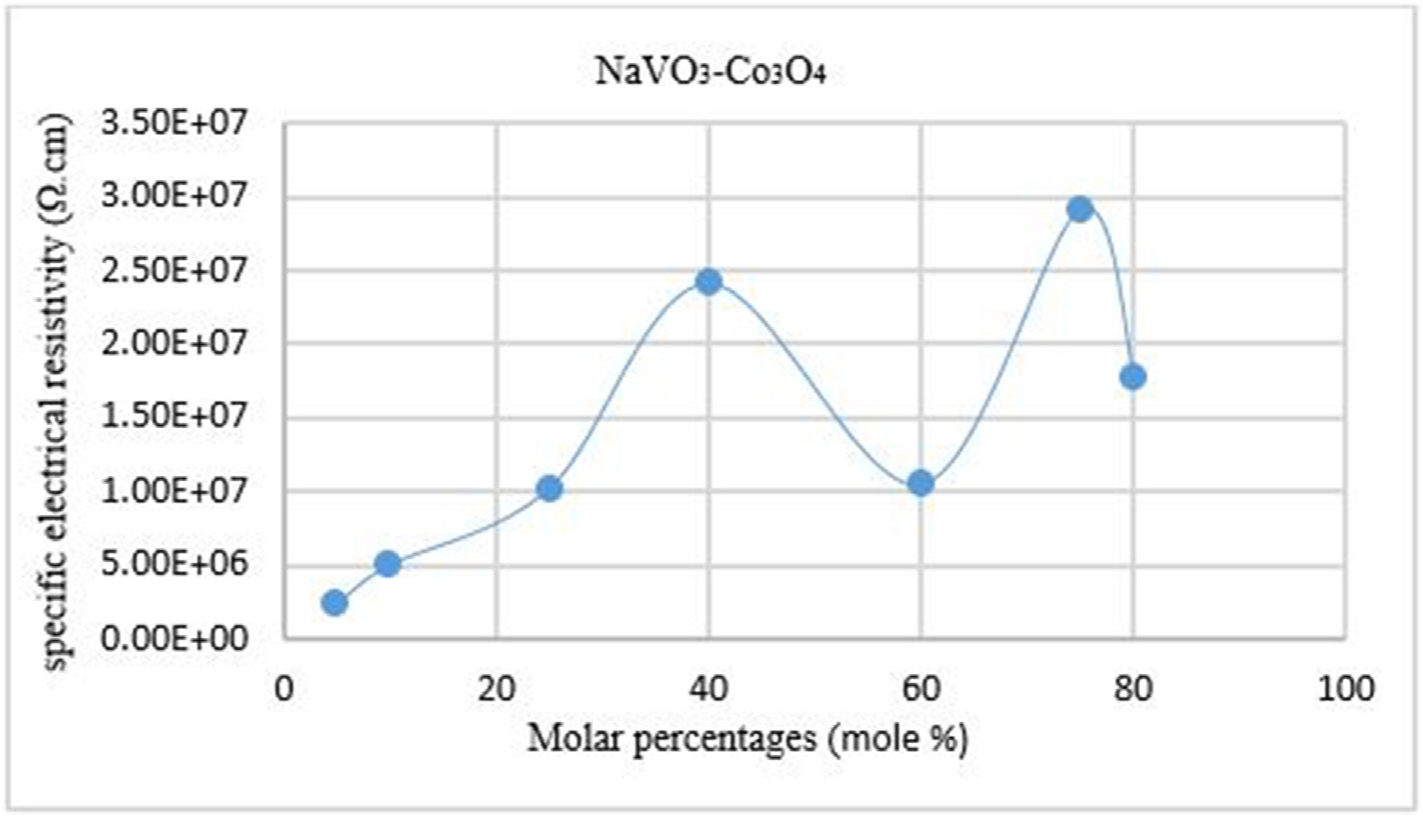
The changes of specific electrical resistivity ρ with the molar percentages function of NaVO_3_.

The results showed a high value of the specific electrical resistivity, it gradually increases with the increase in the percentage of NaVO_3_ in the samples at molar percentages (x = 5, 10, 25, 40). This is due to the change in the crystal structure in this field, where the sodium meta vanadate dissolves and a crystalline solid solution is formed on the basis of cobalt oxide. Then the resistivity value decreases at the molar percentage (x = 60), to return and rise at molar percentages (x = 75, 80). Because of formation of a different crystal structure. This indicates to the different properties of the crystal structure in this field, where it was studied using X−rays diffraction (XRD). According to the equilibrium diagram of the binary system NaVO_3_–Co_3_O_4_. The highest value of the specific electrical resistivity 2.91 × 10^+07^ (Ω.cm) at the molar percentage (x = 75), as shown in Figure 3:

### 3.3 Effect of the frequency f on electrical capacitance C

The electrical capacitance changes C for the prepared samples were studied with Pico farad unit pF according to the binary system _(x)_ NaVO_3_−_(100-x)_Co_3_O_4_ with frequency function f. The results of the study showed that the electrical capacitance values increase with increasing frequency at the molar percentages (x = 5, 10). And it decreases with increasing frequency at the rest of the molar percentages (x = 25, 40, 60, 75, 80).

This indicates that the increase in the molar percentage of sodium meta vanadate in the prepared samples contributed to a decrease in the electrical capacitance values with increasing frequency. The electrical capacitance values change with the change in the percentage of sodium meta vanadate in the samples according to C (x = 60)> C (x = 10)> C (x = 5)> C (x = 40)> C (x = 25). Due to the dissolution of sodium meta vanadate and changing the crystal structure. The electrical capacitance values at these molar percentages range within the field (4.24–5.85) pF. Then the capacitance values increase dramatically with the increase in the percentage of sodium meta vanadate in the prepared samples according to C (x = 75)> C (x = 80). This indicates that the crystal structure is different. The largest value of the electrical capacitance 9.05 pF at the frequency f = 1.00 × 10^+04^ Hz, for the molar percentage (x = 75).

**Figure 4:**
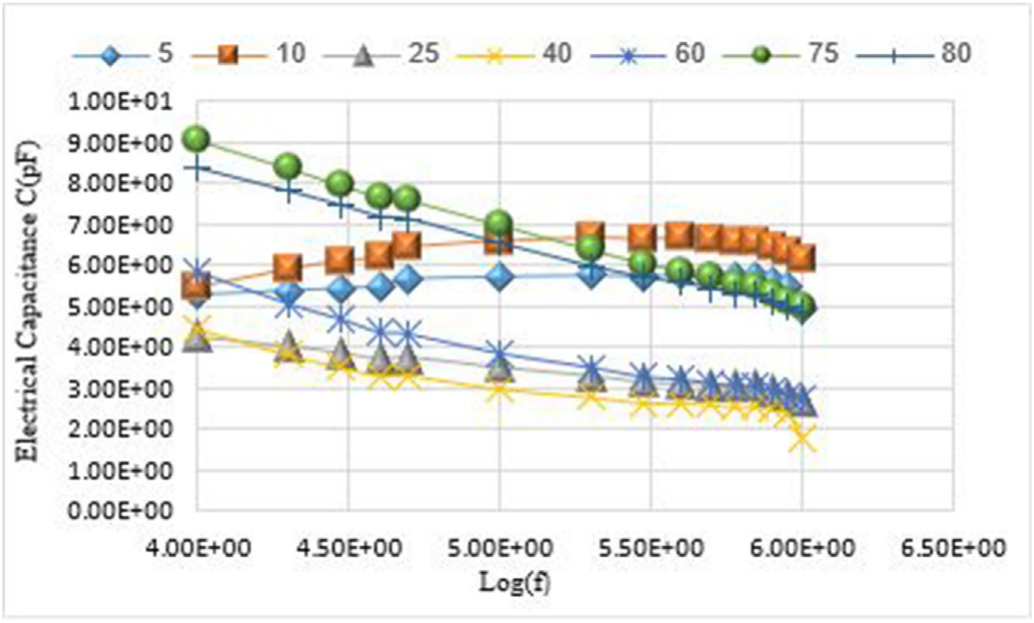
Changes of the electrical capacitance C with frequency dependence f.

Figure 4 shows the changes of electrical capacitance values for the prepared samples, at molar percentages (x = 5, 10, 25, 40, 60, 75, 80) of sodium meta vanadate.

### 3.4 Effect of the frequency f on the relative dielectric constant Ԑ_r_

The capacity of the prepared insulating tablet in the studied binary system _(x)_NaVO_3_−_(100-x)_Co_3_O_4_ is given by:

(1) $$\text{C}={\text{ε}}_{0}{\text{ε}}_{\text{r}}\frac{\text{A}}{\text{d}}$$

From this relationship we get the relative dielectric constant.

When the capacitance is expressed in pF and the geometric dimensions of the tablet in cm (1/ε_o_ = 1/8.85 × 10^−14^ = 11.3 × 10^12^), we find [12]:

(2) $${\text{ε}}_{\text{r}}=11.3\times 10^{12}\;\text{C}\;\frac{\text{d}}{\text{A}}$$

By applying Eqs. (1) and (2), the relative dielectric constant values were obtained. The results showed that the values increase with increasing frequency at the molar percentages (x = 5, 10), and it decreases with increasing frequency at the rest of the molar percentages (x = 25, 40, 60, 75, 80). This indicates that the increase in the molar percentage of sodium meta vanadate in the prepared samples contributed to a decrease in the relative dielectric constant values with increasing frequency. Because of the change in the crystal structure. The values of the relative dielectric constant Ԑ_r_ change with the change in the proportion of sodium meta vanadate in the prepared samples according to Ԑ_r_ (x = 10)> Ԑ_r_ (x = 5)> Ԑ_r_ (x = 40)> Ԑ_r_ (x = 25). The values of the relative dielectric constant Ԑr at these percentages range within the field (11.02–11.76). Then the values of the dielectric constant increase with the increase in the percentage of sodium meta vanadate in the samples according to Ԑ_r_ (x = 75)> Ԑ_r_ (x = 60)> Ԑ_r_ (x = 80). Whereas, the largest value of the dielectric constant is 28.39 at frequency f = 1.00 × 10^+04^ Hz, for the molar percentage (x = 75).

**Figure 5:**
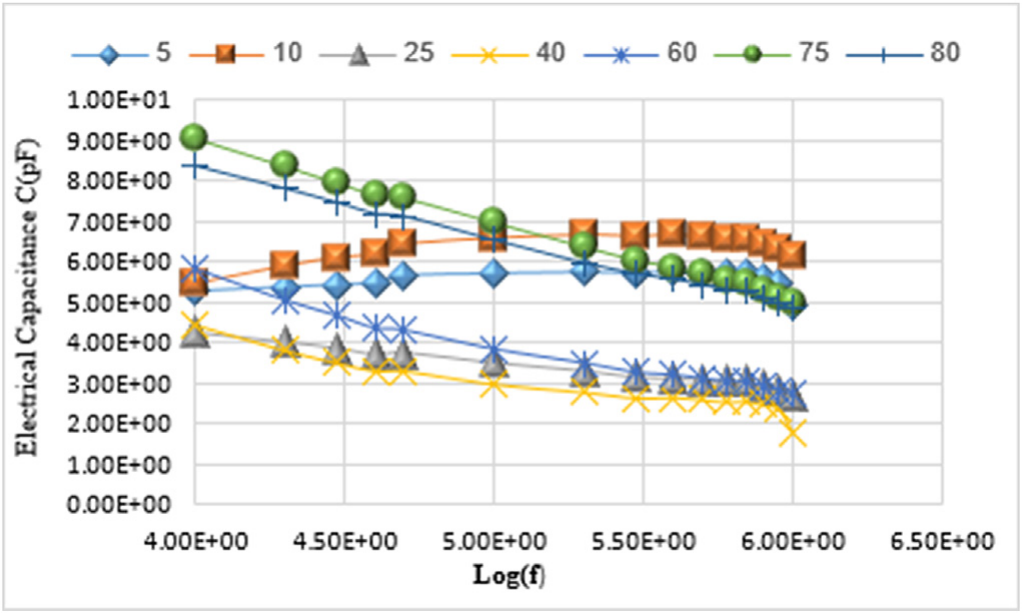
Changes of the of the relative dielectric constant Ԑ_r_ with a frequency dependency f.

Figure 5 Shows the changes in the relative dielectric constant values of the prepared samples at molar percentages (x = 5, 10, 25, 40, 60, 75, 80) of sodium meta vanadate.

### 3.5 Effect of the frequency f on the loss tangent tanδ

tanδ is the amount of energy dissipated in the dielectric. For small values of the loss tangent, the material is a good insulator, and for large values, the energy dissipated in the insulating material is large.

The changes of the loss tangent were studied for the prepared molar percentages in the binary system _(x)_NaVO_3_−_(100-x)_Co_3_O_4_ with the frequency function f according to the relationship:

$$\text{tanδ}=1/\left(\text{ρ}.\text{ω}.\varepsilon_{0}.{\text{ε}}_{\text{r}}\right)$$

where: ω=2πf, angular frequency (rad/sec), f: Frequency (Hz) [13].

The results showed that the loss tangent values for all prepared samples decreases with the increase in frequency, because of the change in the crystal structure. Which indicates that the increase in the molar percentage of sodium meta vanadate in the prepared samples contributed to the decrease in the loss tangent values with the increase in frequency.

Where the lowest value of the loss tangent is 2.17 × 10^−3^, at frequency f = 1.00 × 10^+04^ Hz, for the molar percentage (x = 75). Which corresponds to the highest value of the specific resistivity. In addition, the highest value of the loss tangent is 7.01 × 10^−2^, at frequency f = 1.00 × 10^+04^ Hz, for the molar percentage (x = 5). Which corresponds to the lowest value of the specific resistivity. The values of the loss tangent change with the change in the percentage of sodium meta vanadate in the samples according to:

tanδ(x = 5)> tanδ (x = 10)> tanδ(x = 25)> tanδ (x = 60)> tanδ(x = 40)> tanδ(x = 80)> tanδ (x = 75).

**Figure 6:**
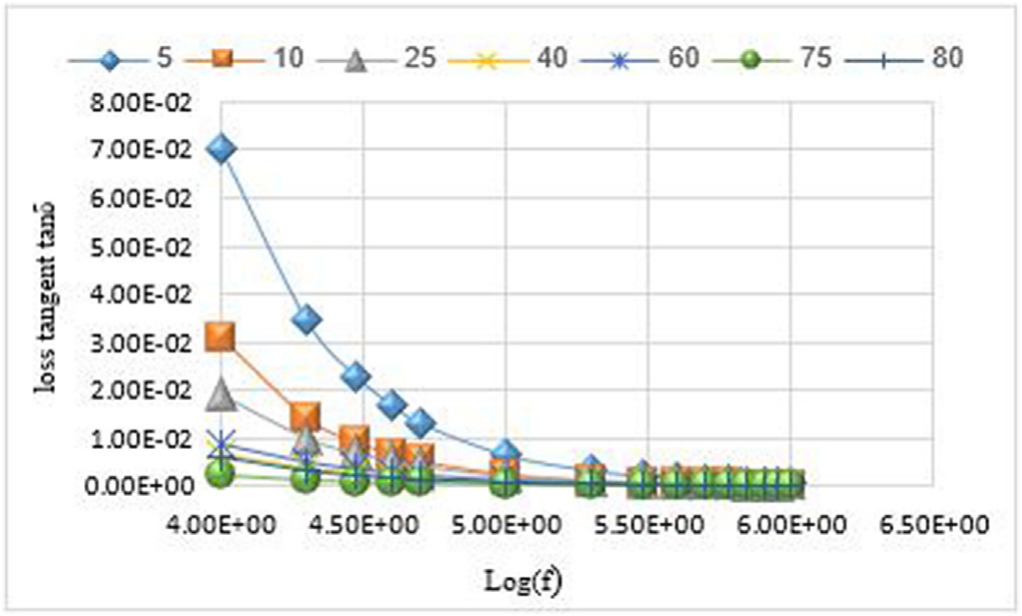
Variations of the loss tangent with the frequency function f.

Figure 6 illustrates the loss tangent changes for the prepared samples at the molar percentages (x = 5, 10, 25, 40, 60, 75, 80).

## 4 Conclusion

### 4.1 Results

• The value of the specific electrical resistivity ρ gradually increased with the increase of the percentage of NaVO_3_ in the samples at the molar percentages. And the highest value of the specific resistivity is 2.91 × 10^+07^ (Ω.cm) at the molar percentage (x = 75).
• The values of the electrical capacitance C change with the change in the percentage of sodium meta vanadate in the samples according to C (x = 60)> C (x = 10)> C (x = 5)> C (x = 40)> C (x = 25). Then the capacitance values increase dramatically with the increase in the percentage of sodium meta vanadate in the prepared samples according to C (x = 75)> C (x = 80). And the largest value for the electrical capacitance is 9.05 pF at frequency f = 1.00 × 10^+04^ Hz of molar percentage (x = 75).
• The values of the relative dielectric constant Ԑ_r_ change with the change in the percentage of sodium meta vanadate in the prepared samples according to Ԑ_r_ (x = 10)> Ԑ_r_ (x = 5)> Ԑ_r_ (x = 40)> Ԑ_r_ (x = 25). Then the values of the dielectric constant increase dramatically with the increase in the percentage of sodium meta vanadate in the samples according to Ԑ_r_ (x = 75)> Ԑ_r_ (x = 60)> Ԑ_r_ (x = 80). The largest value of the dielectric constant is 28.39 at frequency f = 1.00 × 10^+04^ Hz for the molar percentage (x = 75).
• The lowest value of the loss tangent is 2.17 × 10^−3^, at frequency f = 1.00 × 10^+ 04^ Hz for the molar percentage (x = 75). And the highest value of the loss tangent is 7.01 × 10^−2^, at frequency f = 1.00 × 10^+04^ Hz for the molar percentage (x = 5). Where the values of the loss tangent change with the change of the percentage of sodium meta vanadate in the samples according to:

tanδ (x = 5)> tanδ (x = 10)> tanδ (x = 25)> tanδ (x = 60)> tanδ (x = 40)> tanδ(x = 80)> tanδ(x = 75).

### 4.2 Recommendations

Apply of the molar percentages of the prepared samples in the binary system _(x)_NaVO_3_−_(100-x)_ Co_3_O_4_ within batteries in order to develop them and increase their capacity, due to the high values obtained for specific electrical resistivity, electrical capacity, relative dielectric constant, and the loss tangent.

## Declarations

### Author contribution statement

Rasha Hosam Saleh: Conceived and designed the experiments; Performed the experiments; Analyzed and interpreted the data; Contributed reagents, materials, analysis tools or data; Wrote the paper.

Dr. Mohamad Deeb: Analyzed and interpreted the data.

### Funding statement

This work was supported by Tishreen University.

### Data availability statement

Data will be made available on request.

### Declaration of interests statement

The authors declare no conflict of interest.

### Additional information

No additional information is available for this paper.
